# Orbital volume measurement using direct measurement on 3D-printed models: a comparative study

**DOI:** 10.1097/MS9.0000000000005593

**Published:** 2026-10-07

**Authors:** Mathilde De Massary, Pierre Labalette, Matthias Schlund, Benjamin Bouet, Emmanuel Chazard, Romain Nicot

**Affiliations:** a Department of Ophthalmology, University Lille, CHU Lille, Service de d’Ophtalmologie, Lille, France; b Public Health Department, University Lille, CHU Lille, URL 2694 – METRICS, CERIM, Lille, France; c Department of Oral and Maxillofacial Surgery, University Lille, CHU Lille, INSERM, U1008 – Advanced Drug Delivery Systems, Lille, France; d Oral and Maxillofacial Department, University Bordeaux, CHU Bordeaux, INSERM, Inserm U1026 – Bioengineering of Tissues, Bordeaux, France; e Department of Oral and Maxillofacial Surgery, University Lille, CHU Lille, Lille, France; f LaMcube – Laboratoire de Mécanique, Multiphysique, Multiéchelle, UMR 9013, University Lille, CNRS, Centrale Lille, Lille, France

**Keywords:** orbit, orbital imaging, orbital measurements, orbital volume, printing, three-dimensional

## Abstract

**Background:**

Accurate orbital volume (OV) measurement is essential for the management of orbital fractures. However, no consensus exists regarding the optimal method for OV assessment. Manual computed tomography (CT) planimetry remains the most widely used technique in clinical practice.

**Methods:**

This study aimed to develop a direct method for measuring OV using 3D-printed orbital models and to compare it with manual CT planimetry in healthy orbits. Eighteen healthy orbits were analyzed. OV measurements were obtained using two direct-filling techniques on 3D-printed models: sand filling (3D-Orb-1) and water filling (3D-Orb-2). The results were compared with manual CT planimetry (Plani-Orb). Reproducibility was assessed using intraclass correlation coefficients, and agreement between methods was evaluated using linear regression and Bland–Altman analysis.

**Results:**

Mean OV values were 23.34 ± 2.15 cm^3^ for 3D-Orb-1, 23.31 ± 2.17 cm^3^ for 3D-Orb-2, and 24.10 ± 2.37 cm^3^ for Plani-Orb. Reproducibility was excellent for all methods, with ICC values of 0.963, 0.990, and 0.987 for 3D-Orb-1, 3D-Orb-2, and Plani-Orb, respectively. Strong correlations were observed between CT planimetry and both sand-filling (*R*^2^ = 0.823; *P* < 0.0001) and water-filling techniques (*R*^2^ = 0.798; *P* < 0.0001). Bland–Altman analysis demonstrated good agreement between methods.

**Conclusion:**

The 3D-printing-assisted OV measurement method is reliable, reproducible, and cost-effective for orbital volumetric assessment in both clinical and preclinical experimental settings. This technique may serve as a valuable tool for validating computer-assisted OV measurement algorithms and for the *in vitro* evaluation of orbital implants and medical devices.

## Introduction

Measuring orbital volume (OV) remains challenging because of the complex anatomy of the orbit. Nevertheless, accurate assessment of OV and its variations plays an important role in the management of orbital fractures^[^1–4^]^. Even small changes in OV may induce axial displacement of the globe. According to the systematic review by Schlund *et al* (2020), a 1 cm^3^ increase in OV may result in 0.85 mm of early enophthalmos or 0.72 mm of late enophthalmos in post-traumatic settings^[^5^]^. Furthermore, OV measurements obtained from the initial computed tomography (CT) scan have been shown to correlate with the development of late enophthalmos^[^5,6^]^. OV can also be combined with other radiological parameters, such as herniated tissue volume, to calculate the hernia volume ratio used in enophthalmos prediction. OV assessment is therefore an important component of the decision-making process between conservative and surgical management of orbital fractures. In addition, OV may serve as a therapeutic target during orbital reconstruction by guiding implant positioning and design. Accurate preoperative OV measurement is consequently considered clinically relevant for the planning and surgical management of traumatic orbital injuries^[^3,4,7,8^]^.

HIGHLIGHTS

- Direct orbital volume (OV) measurement using 3D-printed models is reliable and highly reproducible.
- Sand- and water-filling techniques show a strong correlation with computed tomography planimetry.
- The proposed method provides a cost-effective approach for direct OV assessment.
- This technique may support the validation of computer-assisted OV algorithms and preclinical implant evaluation.

Despite its clinical importance, no consensus currently exists regarding the optimal method for OV measurement. Several techniques have been described in the literature, including manual planimetry as well as semi-automatic and fully automatic methods. Manual CT planimetry, based on slice-by-slice segmentation, remains the most widely used technique in clinical practice^[^3,4,9^]^.

The earliest methods developed for OV measurement relied on orbital filling techniques using materials such as water and relying on Archimedes’ principle^[^3^]^. Although these direct filling methods remain the historical reference standard, their use is essentially limited to the validation of OV measurement algorithms in healthy orbits. Recently, we developed a direct OV measurement method based on 3D-printed models of healthy human orbits^[^10^]^. Using patient-specific CT data, this technique enables direct physical measurement of OV in a clinically relevant and reproducible manner.

This approach may be particularly useful in preclinical models of secondary enophthalmos reconstruction, allowing optimization of the position and volume of intraorbital implants during surgical planning. Surgeons could, therefore, iteratively adjust implant parameters until the desired reconstruction is achieved. Our previous study demonstrated that the 3D printing-assisted OV measurement method was accurate and reproducible, with results comparable to cadaveric direct measurements^[^10^]^. However, this technique has not yet been compared with computer-assisted OV measurement methods, such as CT planimetry.

The aim of the present study was to compare OV measurements obtained using the 3D printing-assisted method with those obtained using the current clinical reference method, namely manual CT planimetry, in healthy orbits. The objectives were, first, to establish a standardized methodology for OV measurement using 3D-printed models, and, second, to evaluate the accuracy, reproducibility, and correlation between the two techniques.

## Materials and methods

### Patients and data collection

Digital Imaging and Communications in Medicine data were obtained from anonymized craniofacial CT scans acquired at Lille University Hospital with submillimetric slice thickness. All analyzed orbits were healthy and free from orbital pathology, including fractures, tumors, or congenital abnormalities. Eighteen healthy orbits were included in the analysis. The methodology for 3D printing-assisted OV measurement was first established and subsequently compared with computer-assisted OV measurement using CT planimetry.

All procedures performed in this study were conducted in accordance with the ethical principles of the Declaration of Helsinki. According to French legislation, Institutional Review Board approval was not required because of the retrospective, fully anonymized design of the study. In addition, data processing was declared to the French Data Protection Authority (“Commission Nationale de l’Informatique et des Libertés”; declaration no. DEC20-344). The requirement for informed patient consent was waived owing to the retrospective, anonymized nature of the study.

### Computer-assisted planimetry for orbital volume measurement (Plani-Orb)

OVs were measured using computer-assisted manual segmentation with 3D Slicer software (version 4.10.2; Kitware, NY, USA), as previously described^[^6^]^. CT datasets were oriented according to the neuro-ocular plane. Sagittal and coronal planes were positioned perpendicular to the axial plane. Manual segmentation was then performed slice-by-slice on axial images from the orbital roof to the orbital floor using a cursor-based contouring method.

Orbital boundaries were defined according to the method described by Shyu *et al*^[^11^]^: laterally by the zygomatic process of the frontal bone; inferomedially by the anterior lacrimal crest; superomedially by the nasal process of the frontal bone; and superiorly and inferiorly by the orbital rims. The lacrimal canal was excluded from the measurements at its superior opening. Posterior boundaries corresponded to the optic canal and the superior and inferior orbital fissures, which were excluded from segmentation. Sagittal and coronal reconstructions were used to verify segmentation accuracy.

After the completion of the segmentation process, the OV was automatically calculated using the software’s statistical analysis tool and expressed in cm^3^.

### 3D-printing-assisted OV measurement (3D-Orb)

The 3D printing-assisted OV measurement method was adapted from the technique previously described by Piot *et al*^[^10^]^. Three-dimensional models were generated using semi-automatic segmentation with ITK-SNAP software^[^12^]^. This process enabled the creation of a virtual 3D representation of the orbital cavity from CT data. A region of interest was selected and progressively segmented in order to preserve only the anatomical structures corresponding to the orbit. In the present study, the entire bony orbital framework was modeled. A threshold ranging from 50 to 1000 Hounsfield units was applied to isolate osseous structures before exporting the segmentation as an stereolithography file using 3D Slicer software^[^13^]^ (Fig. 1).

**Figure 1.**
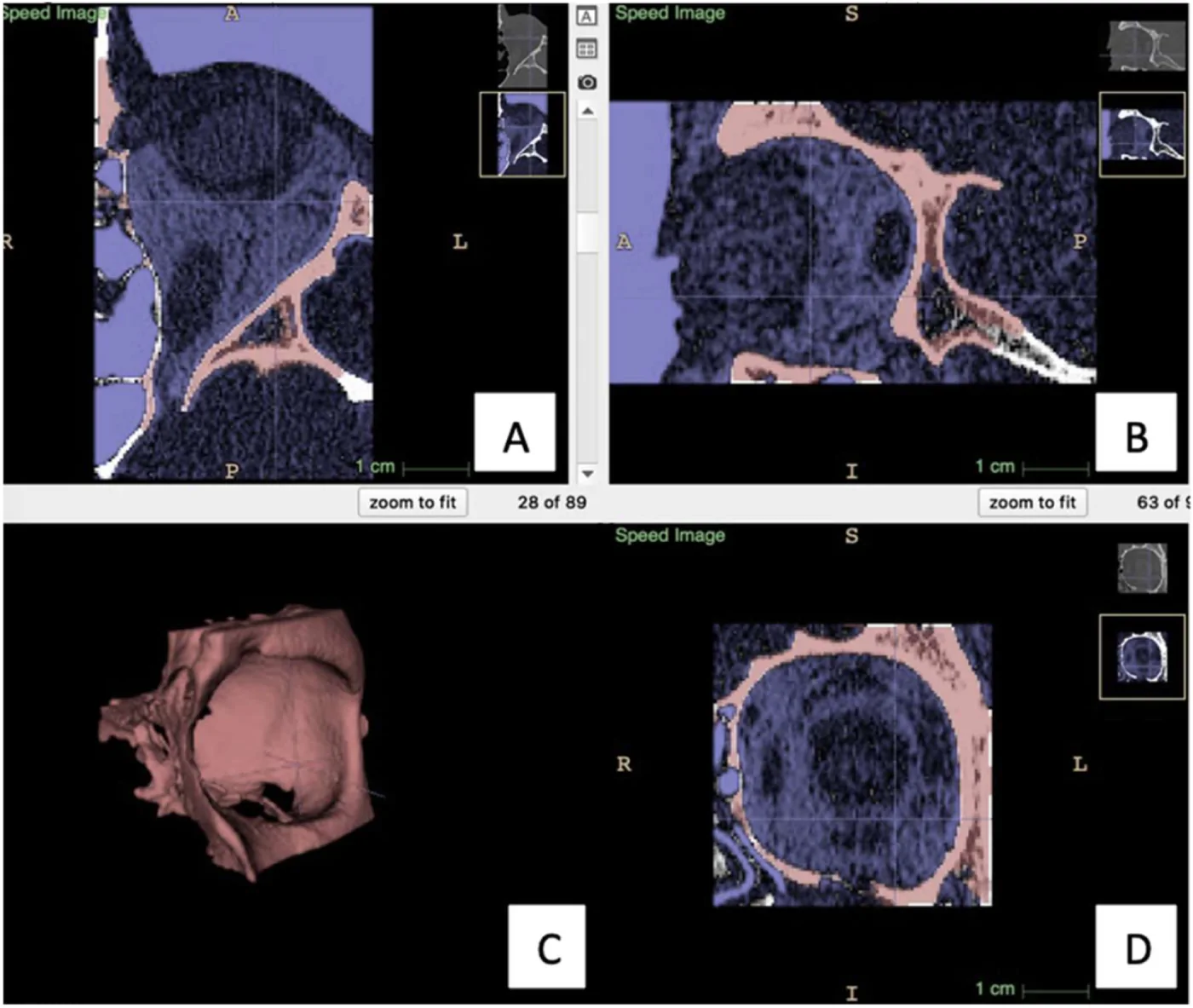
Semi-automatic orbital segmentation using ITK-SNAP software: axial view (A), sagittal view (B), three-dimensional reconstruction (C), and coronal view (D).

The presence of multiple bony openings, including the optic canal, nasolacrimal canal, and orbital fissures, represented a major challenge during the segmentation process. MeshLab software (Visual Computing Laboratory, ISTI-CNR, Italy) and Meshmixer software (Autodesk Inc., San Rafael, CA, USA) were therefore used for post-processing of the 3D models. The “Laplacian Smooth” and “Taubin Smooth” filters available in MeshLab were applied to smooth the orbital surface and remove segmentation artifacts. Subsequently, the “Edit,” “Sculpt,” and “Meshmix” tools in Meshmixer were used to close the various bony openings prior to volume measurement (Fig. 2).

**Figure 2.**
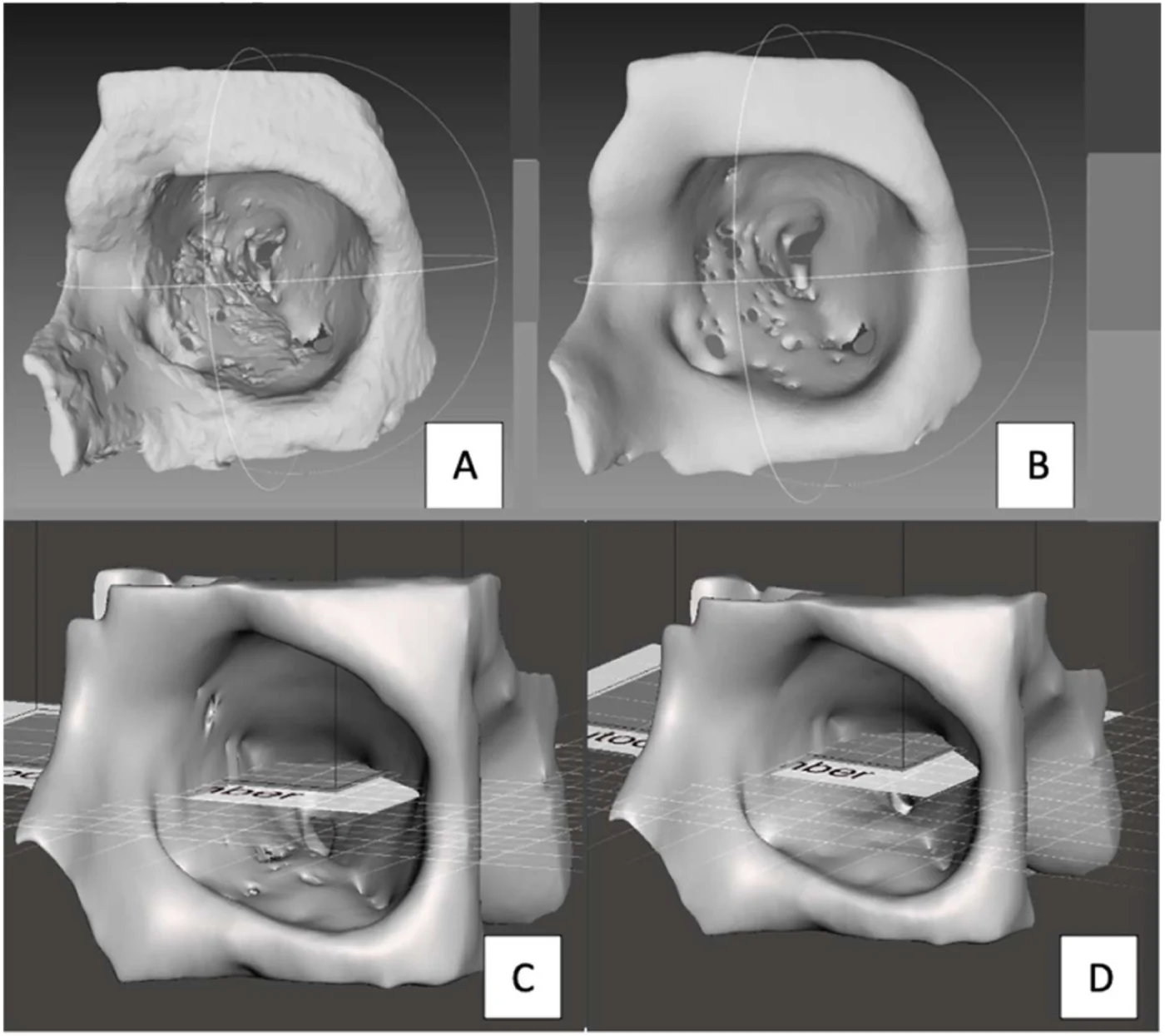
Post-processing of the 3D orbital model using MeshLab and Meshmixer software: application of the “Laplacian Smooth” filter before (A) and after smoothing (B), and the “Taubin Smooth” filter before (C) and after smoothing (D). Closure of bony openings was subsequently performed using Meshmixer tools.

Three-dimensional printing was performed using a UP Plus 2 printer (Beijing TierTime Technology Co. Ltd., Beijing, China). Models were printed in polylactic acid using the following parameters: fast-printing mode, 20% infill density, and a layer thickness of 150 μm. Depending on model complexity and printing settings, the average printing time for a single orbit was approximately 7 hours.

OV was directly measured on the 3D-printed models using two different filling techniques: sand filling (3D-Orb-1) and water filling (3D-Orb-2). Two filling materials with distinct physical behaviors were investigated to evaluate the potential impact of filling characteristics on volumetric accuracy and measurement reproducibility. For the sand-filling method, 0.5-mm-diameter sand particles were poured into the orbital cavity and subsequently transferred into a graduated cylinder for volume determination (Fig. 3). For the water-filling method, water was injected into the cavity using a graduated syringe, allowing direct volumetric measurement. Care was taken to avoid air bubbles during water filling. Each measurement was performed twice independently by two different investigators.

**Figure 3.**
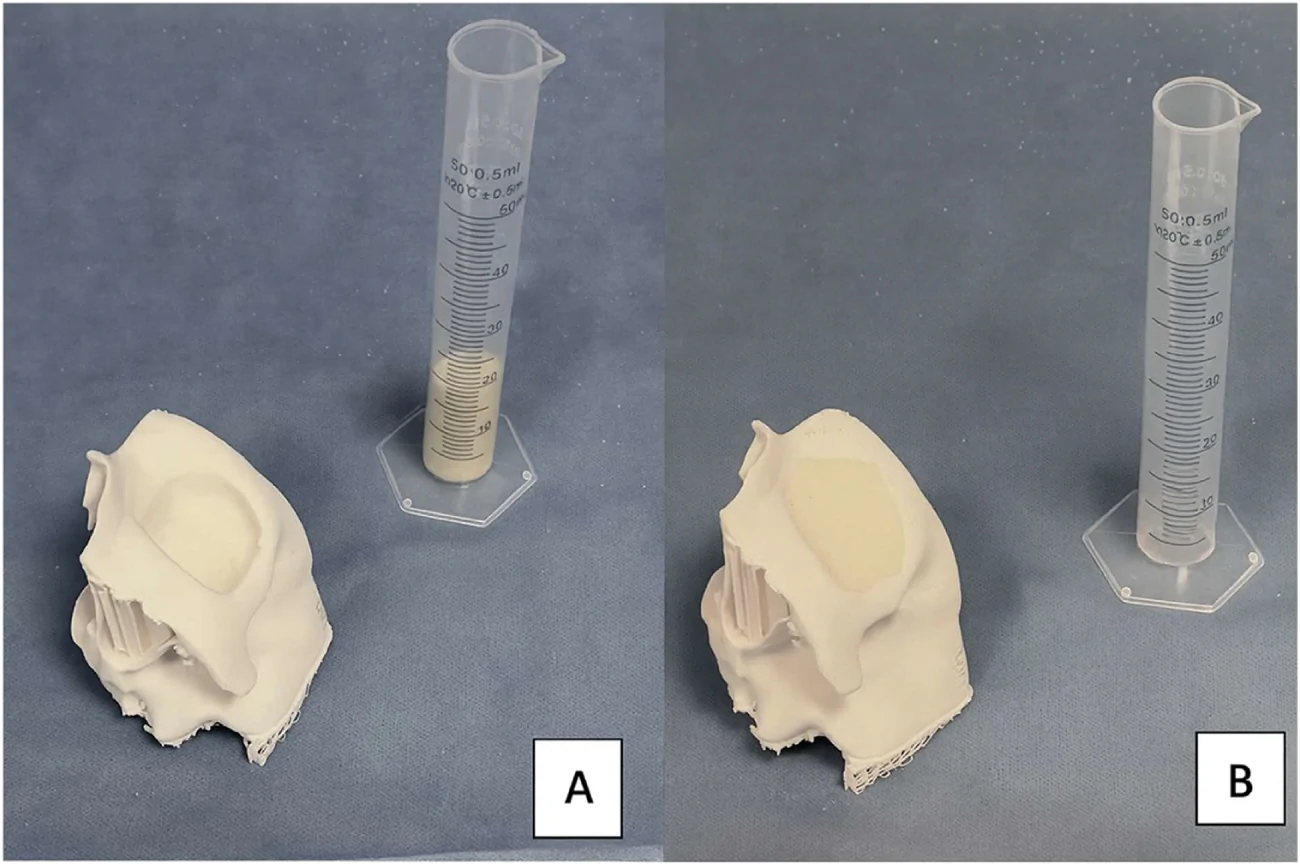
Direct orbital volume measurement on a 3D-printed model. (A) Sand-filling technique using 0.5-mm-diameter sand particles, subsequently transferred to a graduated cylinder for volume determination. (B) Water-filling technique using a graduated syringe.

### Statistical analysis

OVs obtained using the two experimental methods [3D-Orb-1 (sand filling) and 3D-Orb-2 (water filling)] were compared with those obtained using the reference method [Plani-Orb (CT planimetry)].

The normality of the data distribution was assessed using the Shapiro–Wilk test. Descriptive statistics were reported as the mean ± standard deviation (SD).

Intraclass correlation coefficients (ICCs) were calculated to evaluate intraobserver and interobserver reproducibility. Intraobserver reproducibility was assessed for the 3D-Orb-1, 3D-Orb-2, and Plani-Orb methods, whereas interobserver reproducibility was evaluated for the 3D-Orb-1 method. A two-way mixed-effects model with absolute agreement was used to calculate ICCs. ICC values of <0.50, 0.50–0.75, 0.75–0.90, and >0.90 were interpreted as indicating poor, moderate, good, and excellent reproducibility, respectively.

Linear regression analysis was performed to assess the relationship between measurement methods for Observer 1. Correlation strength was interpreted as follows: very weak (0.00–0.19), weak (0.20–0.39), moderate (0.40–0.59), strong (0.60–0.79), and very strong (0.80–1.00).

Agreement between methods was further evaluated using Bland–Altman plots. Limits of agreement were defined as the mean difference ± 1.96 SDs, with SD representing the SD of the differences between measurements.

Statistical analyses were performed using SPSS software, version 25.0 (IBM Corp., Armonk, NY, USA). A *P*-value < 0.05 was considered statistically significant.

## Results

Eighteen healthy orbits were included in the analysis. Descriptive results of OV measurements are summarized in Table 1. Mean OV values were 23.34 ± 2.15 cm^3^ for the 3D-Orb-1 group, 23.31 ± 2.17 cm^3^ for the 3D-Orb-2 group, and 24.10 ± 2.37 cm^3^ for the Plani-Orb group.

**Table 1:** Descriptive statistics of orbital volume (OV) measurements obtained using each measurement method.

Statistic	3D-Orb-1 (sand #1)	3D-Orb-2 (water #1)	3D-Orb-1 (sand #2)	3D-Orb-2 (water #2)	Plani-Orb (observer #1)	Plani-Orb (observer #2)
No. of orbits	18	18	18	18	18	18
Minimum	20	19.5	18.8	19.1	19.919	19.52
Maximum	27.2	27	27.3	27.4	28.32	28.4
1st Quartile	22.225	22.35	22.2	22.2	22.865	22.28
Median	23.15	23.2	23.05	23.2	24.144	23.655
3rd Quartile	24.025	24.1	24.8	24.15	25.287	25.54
Mean	23.339	23.306	23.294	23.128	24.061	23.879
Variance (*n* − 1)	4.635	4.692	6.245	5.736	5.616	6.523
Standard deviation (*n* − 1)	2.153	2.166	2.499	2.395	2.37	2.554

Reproducibility was excellent for all measurement methods, with ICCs of 0.963 for 3D-Orb-1, 0.990 for 3D-Orb-2, and 0.987 for Plani-Orb (Table 2).

**Table 2:** Mean OV values and intraclass correlation coefficients (ICCs) for each OV measurement method.

Measurement Method	Plani-Orb	3D-Orb-1 (Sand)	3D-Orb-2 (Water)
Mean OV (cm^3^)	24.10 ± 2.37	23.34 ± 2.15	23.31 ± 2.17
ICC	0.987	0.963	0.99

A very strong positive correlation was observed between OV measurements obtained using the sand-filling method (3D-Orb-1) and the reference CT planimetry method (Plani-Orb) (*R*^2^ = 0.823; *P* < 0.0001). Similarly, a very strong positive correlation was found between the water-filling method (3D-Orb-2) and Plani-Orb measurements (*R*^2^ = 0.798; *P* < 0.0001). Bland–Altman analysis further demonstrated good agreement between the direct filling techniques and CT planimetry (Fig. 4).

**Figure 4.**
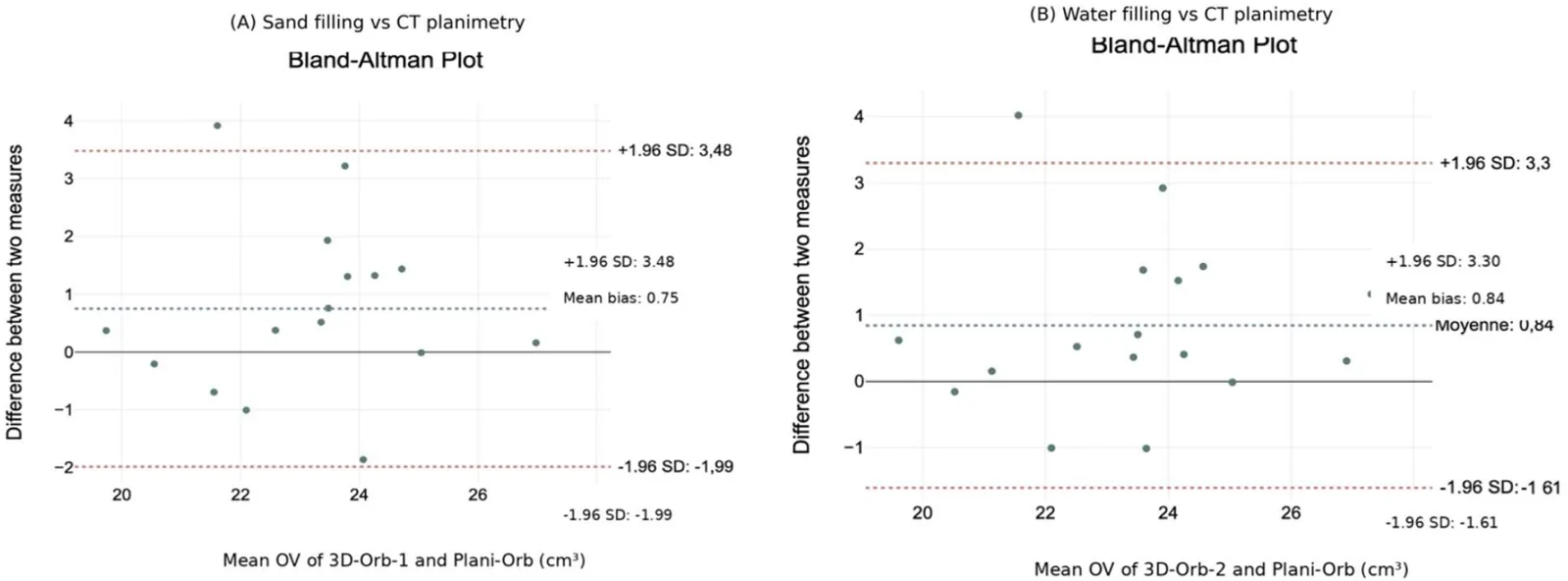
Bland–Altman analyses comparing direct orbital volume measurement techniques with CT planimetry: (A) sand-filling method (3D-Orb-1) versus CT planimetry; (B) water-filling method (3D-Orb-2) versus CT planimetry.

Bland–Altman analysis demonstrated good agreement between the direct filling techniques and CT planimetry. Differences between methods were homogeneously distributed across the range of measured values, without evidence of proportional bias. The limits of agreement remained acceptable for both low and high OV values.

## Discussion

Our study demonstrates that the 3D-printing-assisted OV measurement method is reliable, reproducible, and suitable for both clinical applications and *in vitro* preclinical experimental settings. These findings should be interpreted in light of the anatomical complexity of the orbit and the methodological challenges associated with OV assessment.

Accurate OV measurement remains challenging because of the complex anatomy of the orbit, which contains multiple foramina and fissures. Anatomically, the orbit may be described as a conical structure with the optic canal located at its apex. The anterior opening of the orbit is delineated superiorly and inferiorly by the supraorbital and infraorbital rims and medially and laterally by the anterior lacrimal crest and the frontozygomatic suture, respectively.

The earliest OV measurement techniques relied on direct filling of cadaveric orbits using materials such as lead beads, dry sand, seeds, or water^[^3,14^]^. OV was then calculated from the quantity of filling material required to occupy the orbital cavity. Several direct measurement techniques were subsequently developed using dry skulls and cadaveric models. In 1970, Sarnat^[^15^]^ described an elastic rubber impression technique in a rabbit model and demonstrated, for the first time, the feasibility of cast-based OV measurement, with a reported measurement error of 0.8 cm^3^. Other direct methods were later introduced. Acer *et al*^[^8^]^, for example, described a water-filling technique and found no significant difference compared with CT-based OV measurements. Osaki *et al*^[^16^]^ evaluated several volumetric methods using human cadaveric specimens and concluded that glass bead filling was a simple, reliable, and reproducible technique.

Despite their accuracy, these direct-measurement methods remain limited to cadaveric or experimental settings and cannot be directly applied in routine clinical practice.

To overcome the limitations of direct cadaveric measurements, computer-assisted techniques, such as CT planimetry, have been progressively developed^[^3^]^. The main advantage of manual planimetry is that OV measurement can be performed directly from standard CT imaging without the need for physical models. However, this technique remains operator-dependent and may therefore be associated with interobserver variability.

Gomes de Oliveira *et al*^[^17^]^ demonstrated that manual planimetry provides highly reproducible and accurate OV measurements. Similarly, Jansen *et al*^[^18^]^ reported low intraobserver and interobserver variability values of 0.09 cm^3^ and 0.03 cm^3^, respectively. Nevertheless, both studies emphasized that one of the major methodological challenges remains the definition of the anterior orbital boundary. Kwon *et al*^[^19^]^ further demonstrated that OV measurements may vary depending on the imaging plane used for segmentation and proposed additional anatomical landmarks on axial and coronal views to improve standardization.

In the present study, interobserver reproducibility was excellent for all evaluated methods, with ICC values of 0.963 for the sand-filling technique, 0.990 for the water-filling technique, and 0.987 for CT planimetry. These findings support the reliability of the 3D printing-assisted OV measurement method and suggest that it may represent a clinically relevant adaptation of historical direct measurement techniques originally developed for dry skulls. In addition to potential clinical applications, this approach may also provide a useful preclinical model for evaluating orbital implants and other implantable medical devices used in the correction of enophthalmos. Furthermore, this technique may contribute to patient-specific surgical planning by enabling direct preoperative volumetric assessment and implant simulation.

Piot *et al*^[^10^]^ previously compared the 3D printing-assisted method with cadaveric direct measurements and demonstrated very strong correlations between the two techniques, with excellent intraobserver and interobserver reproducibility (ICCs of 0.997 and 0.998, respectively). However, no comparison with computer-assisted CT planimetry had been performed. To our knowledge, this is the first study to directly compare OV measurements obtained using 3D-printed orbital models with those derived from manual CT planimetry, which remains the current clinical reference method. The present study therefore fills this methodological gap by demonstrating a very strong correlation between OV measurements obtained using the 3D-printed models and those obtained using CT planimetry.

Our findings are consistent with previous studies comparing direct measurement techniques with CT-based volumetric assessment. Cooper^[^14^]^ reported accurate and reproducible OV measurements using CT imaging and sand filling of dry skulls, with differences ranging from 0.2% to 4% between methods. McGurk *et al*^[^20^]^ also demonstrated consistent differences between low-dose CT pixel-counting measurements and direct filling techniques using elastic materials. Similarly, Deveci *et al*^[^21^]^ found no significant differences between CT-based 3D reconstruction software and direct dry skull measurements.

This study has several limitations. The relatively small sample size remains a limitation of this pilot methodological study. Semi-automatic orbital segmentation using ITK-SNAP required substantial manual correction. Because of the thinness of the medial orbital wall and orbital floor, segmentation artifacts frequently created artificial bony defects, even when high-resolution CT datasets were used. As a result, complete delineation of the orbital cavity remained challenging during 3D reconstruction.

Closure of the various bony openings was therefore necessary to prevent leakage of filling material during direct OV measurements. However, this additional post-processing step may have introduced minor volumetric variations. Piot *et al*^[^10^]^ previously demonstrated that computerized closure techniques were slightly less accurate than manual obturation methods, although they offered faster processing and better integration into the digital workflow. In addition, although the technique requires additional segmentation, post-processing, and printing time, material costs remain relatively low and may be acceptable in selected preclinical or personalized surgical planning applications.

Another important limitation, shared by most direct OV measurement techniques^[^7,20,21^]^, concerns the complex geometry of the anterior orbital opening. In all direct-filling methods, the examiner must estimate the curved anterior boundary of the orbit, which may influence volumetric measurements. Osaki *et al*^[^16^]^ reported that glass-bead filling and elastic-molding techniques were more accurate than planimetry because they better reproduced the convex morphology of the anterior orbital surface.

Similarly, one could hypothesize that water-filling techniques, such as CT planimetry, may slightly underestimate OV compared with sand filling because of their limited ability to account for the curved anterior orbital contour. Nevertheless, no significant differences were observed between the measurement techniques in the present study. No clinically relevant systematic bias was identified between direct filling techniques and CT planimetry. In addition, Acer *et al*^[^8^]^ found no statistically significant differences between stereological OV measurements and water-filling techniques in dry skull specimens.

## Conclusion

This study demonstrates that the 3D-printing-assisted technique is a reliable, reproducible, and cost-effective method for OV measurement in both clinical and preclinical experimental settings. Although more time-consuming than automated approaches, it provides a direct physical measurement rather than an estimated volumetric calculation. This technique may, therefore, represent a valuable tool for validating computer-assisted OV measurement algorithms, as well as for the in vitro evaluation of orbital implants and other implantable medical devices.
